# Outcome of stand-alone thoracoscopic epicardial left atrial posterior box isolation with bipolar radiofrequency energy for longstanding persistent atrial fibrillation

**DOI:** 10.1007/s12471-015-0785-3

**Published:** 2015-12-21

**Authors:** M. G. Compier, J. Braun, A. Tjon, K. Zeppenfeld, R. J. M. Klautz, M. J. Schalij, S. A. Trines

**Affiliations:** 1 Department of Cardiology, Leiden University Medical Center, PO Box 9600, Leiden, The Netherlands; 2 Department of Cardiothoracic Surgery, Leiden University Medical Center, Leiden, The Netherlands; 3 Department of Cardio-thoracic Surgery, Onze Lieve Vrouwe Gasthuis, Amsterdam, The Netherlands

**Keywords:** Atrial fibrillation, Surgical ablation, Posterior left atrium, Box isolation

## Abstract

**Introduction:**

Catheter ablation of longstanding (> 1 year) persistent atrial fibrillation (AF) is associated with poor outcome. This might be due to remodelling and fibrosis formation, mainly located in the posterior left atrial (LA) wall. Therefore, we adopted a thoracoscopic epicardial box isolation of the posterior left atrium using bipolar RF energy with intraoperative testing of conduction block.

**Methods and results:**

Bilateral thoracoscopic box isolation was performed with a bipolar RF clamp. Entrance block was defined as absence of a conducted electrogram within the box, while exit block was confirmed by pacing at 10.0 V/2 ms. Ablation outcome was evaluated after 3, 6, 12 and 24 months with 12-lead ECGs and 24-hour Holter recordings.

Twenty-five consecutive patients were included (58 ± 7 years, persistent AF duration 1.8 ± 0.9 years). Entrance block was achieved in all patients and exit block confirmed if sinus rhythm was achieved. After 17 ± 7 months, 76 % of the patients (*n* = 19) were free of AF recurrence. One patient died within 1 month and was considered an ablation failure. Four patients with AF recurrences regained sinus rhythm with additional catheter ablation or antiarrhythmic drugs.

**Conclusions:**

Treatment of longstanding persistent AF with thoracoscopic epicardial LA posterior box isolation using bipolar RF energy with intraoperative testing of conduction block is feasible and highly effective.

## Introduction

Atrial fibrillation (AF) is the most common arrhythmia and associated with increased morbidity and mortality. The pulmonary veins (PVs) have been identified as an important source of AF induction [1]. Treatment aiming at electrical PV isolation is well established for paroxysmal AF. PV isolation alone, however, has been associated with high recurrence rates for persistent AF [2].

Electrical and structural remodelling of the left atrium, which occurs after long episodes of AF, may contribute to the substrate maintaining AF [3]. The extensive surgical Cox-Maze procedure, which combines PV isolation with biatrial substrate modification, has been highly effective in the treatment of persistent and longstanding persistent (duration > 1 year) AF [4]. The procedure requires cardiopulmonary bypass and prolonged aortic cross-clamp times and may therefore be less suitable in case of lone AF.

Recent studies with late gadolinium enhancement-magnetic resonance imaging have shown that atrial fibrosis is mainly found in low-voltage areas of the atrium and is an important component of structural remodelling in AF, which is most prominent in the posterior wall of the left atrium [5]. Low-voltage areas show a decreased conduction velocity and effective refractory period, which leads to the formation of reentry circuits and perpetuation of the arrhythmia [6]. Previous research has found that the presence of low-voltage areas is a predictor for failure of AF ablation and AF recurrence during follow-up [5]. Accordingly, creating a box isolation of the posterior left atrial (LA) wall including the PVs was found to decrease AF recurrences after ablation in patients with paroxysmal and (longstanding) persistent AF [7]. However, minimally invasive stand-alone surgery using unipolar microwave energy aiming at box isolation demonstrated unsatisfying results, probably due to lack of transmurality [8].

The purpose of this study was to evaluate the feasibility of bipolar thoracoscopic epicardial LA box isolation including the PVs, confirmed by intraoperatively assessed bidirectional conduction block in patients with longstanding persistent AF and to evaluate the outcome of this procedure.

## Methods

### Patient population

Consecutive patients with symptomatic drug-refractory longstanding persistent AF between October 2009 and April 2012 who underwent a stand-alone thoracoscopic LA posterior box lesion with intraoperative testing of conduction block were included in this evaluation. At least one type of class 1 or 3 antiarrhythmic drugs had failed and longstanding persistent AF was documented on ECG before surgery. The definition of longstanding persistent AF was based on Heart Rhythm Society/European Heart Rhythm Association guidelines.

Clinical and surgical data were prospectively collected in the departmental Cardiology/Thoracic Surgery Information System (EPD-Vision®, Leiden University Medical Center, Leiden, the Netherlands) and retrospectively analysed. Before surgery, all patients underwent echocardiographic evaluation of the anatomy of the left atrium. Transoesophageal echocardiography was used to exclude the presence of LA thrombus. Antiarrhythmic drugs and oral anticoagulation were discontinued 2–3 days before surgery.

### Surgical procedure

Surgery was performed under general anaesthesia with a double-lumen endotracheal tube.

**Fig. 1:**
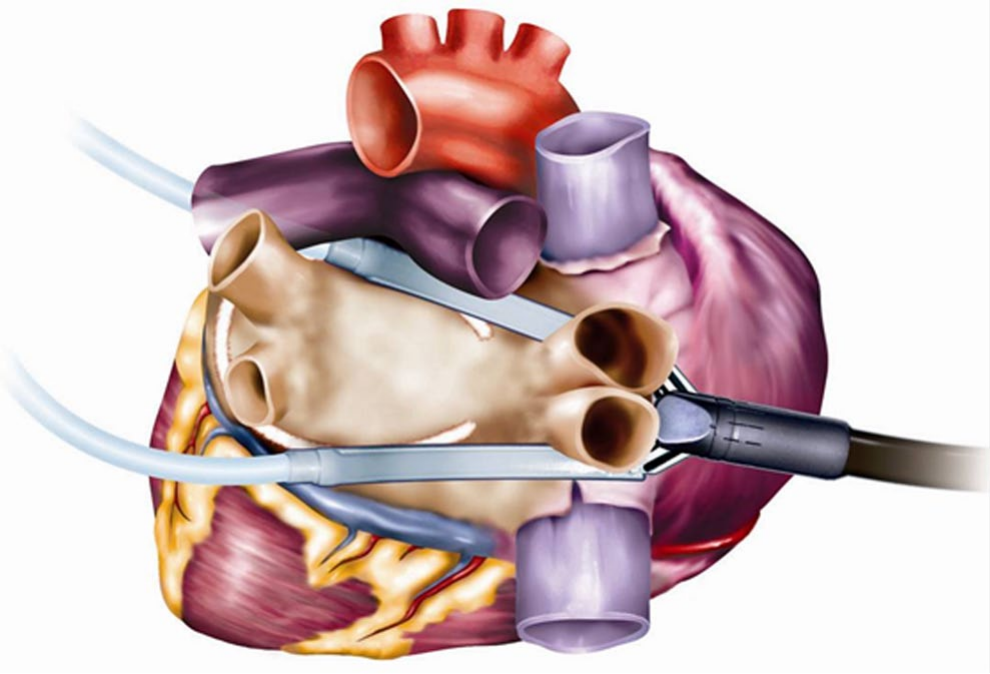
Creation of the box including the left atrial posterior wall and pulmonary veins. (Medtronic Inc., Minneapolis)

Bilateral thoracoscopic chest tubes were inserted. Two guidance catheters were introduced through the oblique and transverse sinuses from the left to the right-sided chest tube as previously described [9]. A bipolar RF clamp (Cardioblate Gemini S, Medtronic Inc., Minneapolis) was attached to the guidance catheters and positioned to encircle the left PVs and posterior left atrium (Fig. 1). Energy delivery was applied 4 times with the convexity of the clamp towards the left atrium and 4 times with the convexity of the clamp facing towards the posterior aspect of the pericardium. Each application was continued until the RF generator indicated transmurality. This is a built-in measurement based on a proprietary algorithm using impedance measurements in the RF-ablation unit. After each application, the jaws of the clamp were repositioned to widen the ablation line. This procedure was repeated on the right side, creating a box that isolates the posterior LA wall including the PVs. Afterwards, electric cardioversion was performed if sinus rhythm was not achieved during the ablation. The left atrial appendage (LAA) was excluded with a 60 mm stapler (Endo GIA Universal Stapler, Covidien Surgical Solutions), unless this was regarded unsafe based on the anatomical and spatial orientation of the LAA, evaluated by the surgeon during the operation.

### Electrophysiological assessment

After identification of the left PVs, bipolar electrograms of the left PVs and posterior left atrium were recorded at maximum gain (0.05 mV/mm using the MAPS device (Medtronic Inc., Minneapolis) before ablation. These signals were used as baseline measurements. The same measurements were performed on the right PVs. After bilateral ablation, recordings were repeated at the same sites with identical amplification to test for entrance block. Entrance block was confirmed by the absence of sharp non-farfield electrograms within the PVs and the box. If sinus rhythm was achieved, bipolar pacing at 10.0 V with 2 ms pulse width was performed from all isolated PVs and from the box to ensure exit block.

### Clinical follow-up

Patients were followed-up at 3, 6, 12 and 24 months after surgery according to the institutional clinical care protocol (MISSION! AF). Evaluation included transthoracic echocardiography (TTE) at 3 and 12 months after the procedure to evaluate LA dimensions and function.

All patients commenced or continued antiarrhythmic drugs until at least 3 months after the procedure. After the first follow-up, antiarrhythmic drugs were discontinued if patients were in sinus rhythm. Oral anticoagulation was resumed and continued for at least 6 months after surgery to prevent thromboembolic complications. Discontinuation after 6 months was at the cardiologist’s discretion and was guided by the CHADS_2_VA_2_SC score, the presence or absence of successful LAA removal and presence of sinus rhythm and LA contraction on echocardiography. Heart rhythm was documented at each visit with ECG and 24-hour Holter recordings. AF recurrence was defined as any recording of an atrial arrhythmia on ECG or an atrial arrhythmia > 30 s on Holter registration after a blanking period of 3 months. Patients were requested to visit the hospital in case of symptoms potentially related to an arrhythmia.

### Statistical analysis

All subsequent patients with longstanding persistent AF undergoing thoracoscopic box isolation were prospectively included. Data were analysed using SPSS (version 20.0, SPSS Inc., Chicago, IL, USA). Continuous data were expressed as mean ± standard deviation. No comparative statistics were performed.

## Results

### Baseline characteristics

Twenty-five patients (76 % male, age 58 ± 7 years) were included. One patient died during the blanking period after the ablation and was included as an ablation failure. Mean duration of longstanding persistent AF was 1.8 ± 0.9 years and the average LA diameter on echocardiography (parasternal long-axis view) was 45 ± 5 mm. Three patients had undergone previous catheter-guided PV isolation performed with cryoenergy (*n* = 1) or radiofrequency (*n* = 2). Other baseline characteristics are shown in Table 1.

**Table 1:** Baseline characteristics

Baseline characteristics	
Male % (*n*)	76 (19)
Age at operation (years, ± SD)	58 ± 7
Body mass index (kg/m^2^, ± SD)	26 ± 3
LA size (mm, ± SD)	45 ± 5
AF duration (years, ± SD)	1.8 ± 0.9
Previous ablations % (*n*)	12 (3)
Structural heart disease % (*n*)	
- Mitral valve disease	4 (1)
- Aortic valve disease	8 (2)
- Cardiomyopathy	4 (1)
Coronary artery disease % (*n*)	12 (3)
Hypertension % (*n*)	28 (7)
Diabetes mellitus % (*n*)	4 (1)
AADs before surgery (*n*, ±SD)	2 ± 1
CHA_2_DS_2_-VASc (score, ±SD)	1.2 ± 1

*AAD* antiarrhythmic drug, *AF* atrial fibrillation, *LA* left atrium, *SD* standard deviation.

### Procedural outcome

Entrance block was achieved in all patients. No additional RF applications after the initial isolation of the box and PVs were needed. The average duration of a left-sided application was 36 ± 5 s and 33 ± 8 s for a right-sided application. In 20 patients, exit block could be confirmed. In 6 patients (24 %) spontaneous conversion to sinus rhythm occurred during ablation and in 14 patients (56 %) after electrical cardioversion. In the remaining 5 patients (20 %), peri-procedural sinus rhythm was not achieved despite performing electrical cardioversion with a maximum of 3 different attempts. In 12 patients, exclusion of the LAA was deemed safe by the surgeon and the LAA was thus removed. In the other 13 patients, the LAA was not excluded. Other procedural characteristics are shown in Table 2. Conversion to thoracotomy was not required in any of the patients. Twenty patients (80 %) were in sinus rhythm immediately after surgery. In three additional patients, cardioversion was performed during the hospital stay and one patient converted spontaneously to sinus rhythm. Only one patient had AF at hospital discharge, but converted to sinus rhythm within a month. After the thoracoscopic procedure, one patient required thoracoscopic surgical evacuation of pericardial fluid due to a tamponade, one patient experienced delirium and one patient pericarditis and transient neuropraxia of the brachial plexus during hospitalisation.

**Table 2:** Procedural characteristics

Procedural characteristics	
Ablation time (min, ± SD)	9.3 ± 1.7
Procedure time (min, ± SD)	223 ± 54
Entrance block box % (*n*)	100 (25)
Exit block box % (*n*)	80 (20)
Electrocardioversion % (*n*)	76 (19)
SR achieved during surgery % (*n*)	80 (20)

*SD* standard deviation, *SR* sinus rhythm.

### Clinical follow-up

**Fig. 2:**
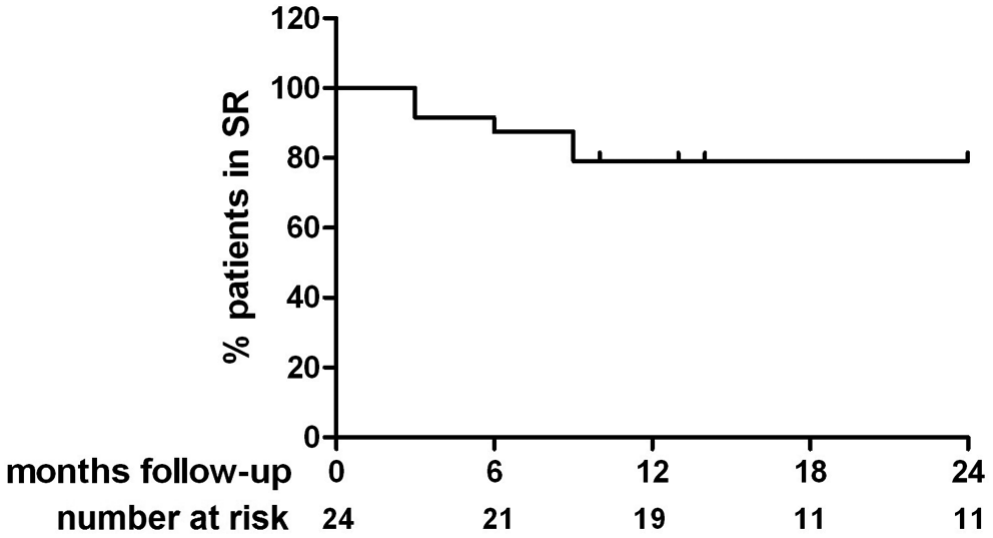
Survival curve of sinus rhythm maintenance during follow-up. *SR* sinus rhythm

After a mean follow-up of 17 ± 7 months (with a minimum of 7 months), 19 out of 25 patients (76 %) were free from any AF recurrence after a 3-month blanking period without the usage of antiarrhythmic drugs (Fig. 2). One patient died during the blanking period due to a cerebrovascular accident. The ablation in this patient was registered as a failure since his death was possibly related to the procedure. Two patients were lost-to-follow-up after the 6-month follow-up appointment. Therefore, 22 patients were included for the 12-month follow-up.

Five patients had recurrences of an atrial arrhythmia. Three patients underwent additional catheter ablation for paroxysmal AF. Two of these patients were found to have incomplete isolation of the box and PVs. In the first patient, the gaps were located in the roofline of the box close to the right superior pulmonary vein and in the inferior line. The second patient had gaps located along the roofline and inferior line of the box. Re-isolation was achieved in one patient, leading to sinus rhythm during follow-up, but not in the other patient, who continued to have AF recurrences after two additional catheter-guided procedures. The third patient had sustained isolation of the PVs and box and regained sinus rhythm after fractionated electrogram ablation and isolation of the superior vena cava. Finally, one patient experienced atypical flutter and one patient paroxysmal AF, which were both successfully treated with flecainide. After 12 months, 12 patients were still on oral anticoagulation.

TTE was performed in 22 patients before the procedure and at 3 and 12 months follow-up to evaluate LA function. Twenty patients (91 %) showed an apparent A-wave during follow-up, representing restoration of atrial contraction. Of the two patients without an A-wave, one patient had persistent AF recurrences despite multiple additional catheter ablations.

## Discussion

This study evaluates the efficacy of thoracoscopic LA box isolation with intraoperative measurement of conduction block for the treatment of longstanding persistent AF. The main findings of this study are: (1) LA posterior box isolation is highly effective in the treatment of longstanding persistent AF and (2) intraoperative testing of isolation of the box and the pulmonary veins is feasible using a pacing and sensing device.

### Catheter-guided ablation

Catheter-guided PV isolation of (longstanding) persistent AF has led to high recurrence rates. Whether an ablation is effective seems to depend on the underlying mechanism that initiates and maintains the arrhythmia, which may differ for each individual AF patient [10]. Especially in patients with (longstanding) persistent AF, multiple factors seem to play a role in perpetuating AF including a trigger initiating the arrhythmia often located in the PVs, arrhythmogenic substrates in the LA posterior wall and conduction pathways at the PV-left atrium transmission side. Ablation may therefore be most effective when all the mentioned factors are modified during the procedure.

In order to increase efficacy of the procedure in patients with paroxysmal and (longstanding) persistent AF, new approaches were proposed including the usage of a new energy source or expanding the ablation procedure. The first results of PV isolation using an endoscopic laser balloon in 50 patients with paroxysmal or persistent AF were recently reported [11]. After a median follow-up period of 17 months, 58 % of patients were in sinus rhythm. These results were in line with or slightly lower than the efficacy reported for PV isolation performed with radiofrequency energy or with the cryoballoon. In an attempt to increase the efficacy of left atrial radiofrequency ablation in patients with longstanding persistent AF, defragmentation was performed during ablation [12]. Defragmentation is electrogram based and targets specific regions in the atrium that show complex fractionated electrograms (CFAEs), which represent areas with a shorter cycle length and local re-entrant wavefronts. The ablation aimed at slowing the cycle length or elimination of the CFAEs. Patients with longstanding persistent AF that underwent ablation with defragmentation (*n* = 20) were compared with a matched control group undergoing ablation without defragmentation (*n* = 17). No difference was found in efficacy after 12 months of follow-up and the ablation with defragmentation had a higher incidence of minor complications.

Finally, the addition of extra ablation lines may improve ablation efficacy. Catheter-guided isolation of the PVs and the posterior left atrium, creating a box, can be performed with an irrigated tip radiofrequency catheter and was found to be feasible in patients suffering from paroxysmal or (longstanding) persistent AF [13, 14]. Results of different studies are represented in Table 3 [15–17]. These studies show that catheter-guided box isolation is feasible, but has limited efficacy in patients with longstanding persistent AF, since sinus rhythm is achieved in less than 50 % of patients at 1–2 years of follow-up compared with 76 % in our study [15–17].

**Table 3:** Studies including patients with (longstanding) persistent atrial fibrillation treated with catheter-guided box isolation

Study	Number of patients	Follow-up period (months)	% SR maintenance after 1 procedure	% SR maintenance after > 1 procedure
Sanders et al. [15]	27	24	44	63
Chen et al. [16]	24	12	50	–
Kumagai et al. [17]	24	12	46	–

*SR* sinus rhythm.

The problem possibly encountered with catheter-guided box isolation is a potential lack of transmural and contiguous lesions, since the lines are created by unipolar point-by-point RF applications. Indeed, recurrences were often caused by conduction gaps in the ablation lines [17].

### Surgical ablation

Several approaches have been suggested to simplify complex and extensive surgical ablation procedures by limiting the number of ablation lines. Restricting the ablation lines to the area of the left atrium which is most affected by structural remodelling, the posterior LA wall, is appealing. Creation of a box including the posterior LA wall and PVs during mitral valve surgery in patients with (longstanding) persistent AF resulted in AF-free survival of 71 % after 6 years follow-up [9].

A minimally invasive approach can also reduce the invasiveness of surgical ablation, especially for patients with lone AF. Video-assisted thoracoscopic bilateral PV isolation was found to be feasible and safe [18].

Thoracoscopic box isolation performed with unipolar microwave energy did not lead to isolation of the box and long-term maintenance of sinus rhythm [8]. Unipolar energy may lead to the absence of transmurality of the created lesions [19]. Incomplete lesions are a risk factor for AF recurrence and induction of other atrial arrhythmias [20]. Ablation with a bipolar energy source is therefore a favourable option, since the energy is directed through the atrial tissue between the clamps, increasing the likelihood of transmurality. Besides this, intraoperative testing of conduction block during this procedure is feasible and can confirm transmurality. The latter was confirmed by Krul et al. who performed a minimal invasive hybrid surgical ablation procedure under electrophysiological guidance in a group of patients with paroxysmal and a group with (longstanding) persistent AF [21]. Patients with persistent AF (*n* = 36) underwent PV isolation with additional LA ablation lines until bidirectional block was confirmed. This procedure was found to be feasible and showed that 75 % of patients with persistent AF were in sinus rhythm at 12-month follow-up.

Altogether, this study showed that performing a thoracoscopic epicardial box isolation using bipolar RF energy is feasible and all patients obtained intraoperative electrical isolation of the box, resulting in high success rates at 17 ± 7 month follow-up.

## Left atrial function and thromboembolic risks

Besides obtaining sinus rhythm, ablation aims at restoration of LA contraction. In this study, 20 out of 22 patients (91 %) showed restoration of atrial contractions. One patient had AF recurrences, which may have caused the absence of atrial contractions. Only one patient maintained sinus rhythm without atrial contractions. Previous research has shown that atrial contraction after surgical AF ablation is not always restored [22]. Only 38 % of patients with longstanding persistent AF showed restoration of LA contraction after a Cox-MAZE IV procedure. Absence of atrial contraction after this type of procedure led to a 5-fold increase in the risk for stroke [23]. The high percentage of restoration of atrial contraction found in this study may therefore indicate a reduced thromboembolic risk compared with the risk before ablation. After the thoracoscopic procedure, one patient died within a month due to a cerebrovascular accident. This patient had a contraindication for anticoagulation due to a subarachnoid bleed in the past. Thromboembolic risk can be reduced by performing surgery under vitamin K antagonist treatment and exclusion of the LAA, which is a thrombogenic structure. During this study, the LAA was excluded whenever possible. LAA exclusion can be contraindicated during a thoracoscopic procedure for anatomical reasons however [24]. In the patient who died after a stroke it was considered unsafe to remove the LAA. Considering thromboembolic risks, the procedure therefore seems to be safe only when performed in patients under vitamin K antagonist treatment.

## Limitations

Although most patients included in this study suffered from longstanding persistent AF, 5 patients only experienced continuous AF for less than 12 months with a minimum of 7 months. The AF duration was not normally distributed with a range from 7 to 48 months and an interquartile range of 14.5. We therefore evaluated whether a duration of persistent AF < 12 months led to a higher incidence of sinus rhythm during follow-up. The outcome for both subgroups (persistent AF duration < 12 months versus persistent AF duration > 12 months) was compared and was found to be not statistically different (*p* = 0.82). Also, the left atrium was only mildly dilated. The left atria were therefore relatively healthy for a longstanding persistent AF cohort.

The first 9 procedures were performed as a minimally invasive thoracotomy instead of a thoracoscopic approach which was used in the other patients. During this procedure, the incisions made to obtain access to the chest were 6–7 cm instead of 1 cm. This increased the invasiveness of the procedure, which may have led to a higher risk of complications. The number of complications was equally distributed between the two procedures, however with 1 complication reported after a thoracotomy (*n* = 9) and 2 after the thoracoscopic approach (*n* = 16).

Two patients were lost-to-follow-up after the 6-month follow-up appointment despite multiple attempts to contact these patients and thus did not complete the 12-month follow-up period. The average follow-up period was therefore shorter than what may have been expected if the entire follow-up had been completed in these patients as well.

This study was single-centre and retrospective. Only a small number of patients were included, without comparison with a matched control group. We therefore cannot substantiate that the box isolation performed during this study is superior to PV isolation alone. Also, 3 patients underwent a previous catheter-guided ablation before surgery. The cumulative effect of both procedures is unknown, so the durability of the box isolation in these patients may have been ameliorated by the previous ablation procedure.

Since catheter-guided ablation has evolved in the last years, a randomised controlled trial comparing surgical with catheter-guided box isolation should be performed to confirm the results found in this study and to evaluate which procedure is more effective and safe.

## Conclusion

Treatment of longstanding persistent AF with thoracoscopic epicardial LA posterior box isolation using bipolar RF energy and with intraoperative testing of conduction block is feasible, highly effective and safe. Left atrial function was found to be restored in the majority of patients, indicating a reduced risk for thromboembolic complications.

### Funding

No external funding was received to perform this research.
